# Poly[diaqua­(μ_4_-benzene-1,2,4,5-tetra­carboxyl­ato)[μ_2_-1,4-bis­(3-pyridyl­meth­yl)piperazine]dizinc(II)]

**DOI:** 10.1107/S1600536811018721

**Published:** 2011-05-25

**Authors:** Hui-Feng Zhang, Yan Li, Wen-Xiu Zhao, Liang-Zhong Zhao, Duo Zhang

**Affiliations:** a College of Life Science, Jilin University, Changchun 130022, People’s Republic of China; b Faculty of Pharmacy, Jilin Medical College, Jilin 132013, People’s Republic of China; c Department of Etiology, Jilin Medical College, Jilin 132013, People’s Republic of China

## Abstract

In the title compound, [Zn_2_(C_10_H_2_O_8_)(C_16_H_20_N_4_)(H_2_O)_2_]_*n*_, the Zn^II^ atom is in a distorted tetra­hedral environment, being coordinated by one N atom from a 1,4-bis­(3-pyridyl­meth­yl)piperazine (3-bpmp) ligand, two O atoms from two carboxyl­ate groups of the pyromellitate anion and one water mol­ecule. The distortion of the tetrahedral coordination may be ascribed to the hydrogen bonds between the carboxyl­ate groups and the adjacent water mol­ecules. Each Zn^II^ atom links to three organic ligands and each pyromellitate ligand coordinates to four Zn^II^ atoms, forming a (3,4)-connected infinite three-dimensional framework. O—H⋯N inter­actions also occur.

## Related literature

For a coordination polymer containing 3-bpmp, see: Martin *et al.* (2009 ▶). For the preparation of *N*,*N*-bis­(3-pyridyl­meth­yl)piperazine, see: Pocic *et al.* (2005 ▶). 3-bpmp and its derivatives are important heteroaromatic *N*-donor bridging ligands for the construction of coordination polymers, see: Farnum & LaDuca (2010 ▶).
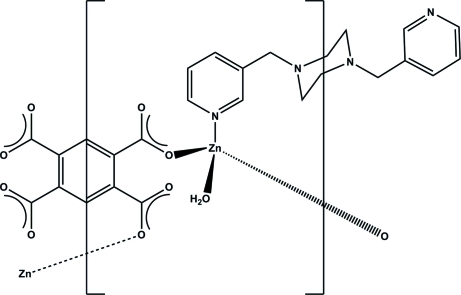


## Experimental

### 

#### Crystal data

[Zn_2_(C_10_H_2_O_8_)(C_16_H_20_N_4_)(H_2_O)_2_]
*M* *_r_* = 685.24
Monoclinic, 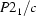

*a* = 9.5630 (5) Å
*b* = 9.8747 (5) Å
*c* = 16.1808 (7) Å
β = 120.673 (2)°
*V* = 1314.20 (11) Å^3^
*Z* = 2
Mo *K*α radiation
μ = 1.89 mm^−1^
*T* = 293 K
0.22 × 0.16 × 0.15 mm

#### Data collection

Bruker SMART APEXII CCD diffractometer
Absorption correction: multi-scan (*SADABS*; Bruker, 2004 ▶) *T*_min_ = 0.672, *T*_max_ = 0.767
7908 measured reflections
3096 independent reflections
2622 reflections with *I* > 2σ(*I*)
*R*_int_ = 0.027

#### Refinement

*R*[*F* ^2^ > 2σ(*F* ^2^)] = 0.028
*wR*(*F* ^2^) = 0.071
*S* = 1.03
3096 reflections
190 parameters
H-atom parameters constrained
Δρ_max_ = 0.44 e Å^−3^
Δρ_min_ = −0.44 e Å^−3^

### 

Data collection: *APEX2* (Bruker, 2004 ▶); cell refinement: *SAINT* (Bruker, 2004 ▶); data reduction: *SAINT*; program(s) used to solve structure: *SHELXTL* (Sheldrick, 2008 ▶); program(s) used to refine structure: *SHELXTL*; molecular graphics: *SHELXTL*; software used to prepare material for publication: *SHELXTL* and local programs.

## Supplementary Material

Crystal structure: contains datablocks I, global. DOI: 10.1107/S1600536811018721/zk2007sup1.cif

Structure factors: contains datablocks I. DOI: 10.1107/S1600536811018721/zk2007Isup2.hkl

Additional supplementary materials:  crystallographic information; 3D view; checkCIF report

## Figures and Tables

**Table:** 

Zn1—O5	1.9442 (16)
Zn1—O1	1.9762 (15)
Zn1—O3^i^	1.9795 (14)
Zn1—N1	2.0443 (18)

**Table:** 

O5—Zn1—O1	121.11 (7)
O5—Zn1—O3^i^	106.68 (7)
O1—Zn1—O3^i^	103.27 (6)
O5—Zn1—N1	111.84 (8)
O1—Zn1—N1	104.45 (7)
O3^i^—Zn1—N1	108.74 (7)

Symmetry code: (i) 
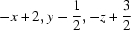
.

**Table 2:** Hydrogen-bond geometry (Å, °)

*D*—H⋯*A*	*D*—H	H⋯*A*	*D*⋯*A*	*D*—H⋯*A*
O5—H5*A*⋯O4^ii^	0.85	1.82	2.662 (2)	173
O5—H5*B*⋯N2^iii^	0.85	1.91	2.751 (2)	169

Symmetry codes: (ii) 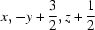; (iii) 
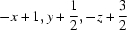
.

**Fig. 1.**
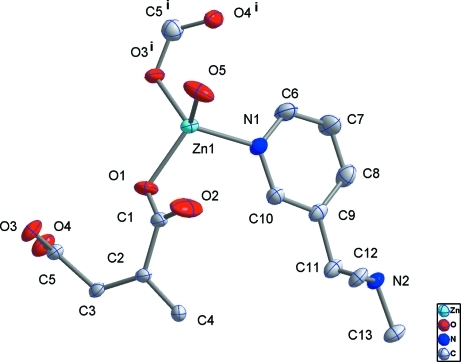
ORTEP drawing of the title components with thermal ellipsoids at the 50% probability level. Symmetry codes: (i) 2 - x, -1/2 + y, 1.5 - z

**Fig. 2.**
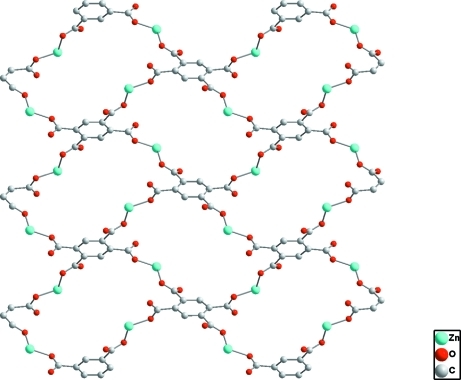
Face-on view of the (4,4)-grid layer motif in the title compound viewing along the c axis.

**Fig. 3.**
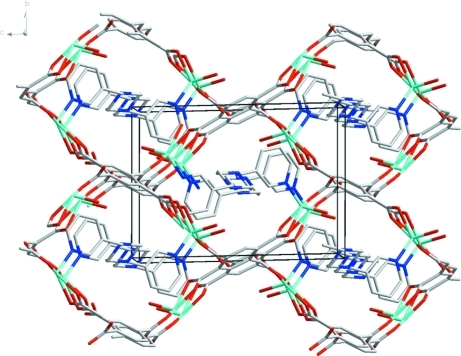
A packing view of three-dimensional network of the title compound nearly viewing along the a axis.

## supplementary crystallographic information

### Comment

1,4-bis(3-pyridylmethyl)piperazine (3-bpmp) and its derivatives are important heteroaromatic N-donor bridging ligands for the construction of coordination polymers (Farnum & LaDuca, 2010). Whereas, only a handful of polymers based on 3-bpmp have been described (Martin *et al.*, 2009). The title compound was prepared during an attempt to prepare a coordination polymer containing both pyromellitic acid and 3-bpmp ligands.

The asymmetric unit of the title compound (Fig. 1) contains a zinc^II^ ion, a half 3-bpmp ligand, a half pyromellitate anion on the crystallographic inversion centre, and one coordinated water molecule. the Zn1 atom is situated in a distorted tetrahedron center, coordinated by one N atoms (N1) from 3-bpmp, two O atoms (O1, O3i) from two carboxylate groups of pyromellitate and one water molecule (Table 1). The other two carboxylate O atoms (O2, O4i) are also close to the Zn1 center, however, the lengths of Zn1—O (2.730 (2) Å) and Zn1—O4i (2.884 (2) Å) are too long to be considered as bonding interactions, thus the Zn^II^ center can be best described as four rather than six coordinated, which may be ascribed to the hydrogen bonds between the carboxylate groups and the adjacent water molecules (Table 1).

In the crystal structure, each pyromellitate ligand coordinates to four Zn^II^ atoms in tetra-monodentate bridging mode, and each Zn^II^ atom is linked to two pyromellitate ligands to construct waved (4,4)-grid [Zn_2_(pyromellitate)]_n_ layers that are oriented parallel to the (1 0 1) crystal planes (Fig. 2). The two Zn^II^ atoms between the adjacent layers are connected by 3-bpmp ligand thus a (3,4)-connected infinite three-dimensional framework are formed (Fig. 3). Withal, there are intralayer hydrogen bonding between the water molecule ligands and unligated pyromellitate O atoms provides additional stabilization of the layer motifs.

### Experimental

Zinc nitrate hexahydrate and pyromellitic acid were obtained commercially. 1,4-bis(3-pyridylmethyl)piperazine was prepared *via* a published procedure (Pocic, *et al.*, 2005). A mixture of zinc nitrate hexahydrate (135 mg, 0.50 mmol), pyromellitic acid (62 mg, 0.25 mmol), 1,4-bis(3-pyridylmethyl)piperazine (33 mg, 0.12 mmol) and 10.0 g water (550 mmol) was placed into a 23 ml Teflon-lined Parr Acid Digestion bomb, which was then heated under autogenous pressure at 398 K for 72 h, then cooled to RT at a rate of 5 °c/h. The resulting yellowish crystals of the title compound were obtained. Elemental analysis (%) calcd for title compound: C, 45.57; H, 3.82; N, 8.18; found: 45.63; H, 3.87; N, 8.23.

### Refinement

All H atoms bound to C atoms were placed in calculated positions, with C—H = 0.93Å (CH) or C—H = 0.95Å (CH2)), *U*_iso_(H) = 1.2 times *U*_eq_(C). The H atoms bound to water molecule O atoms were found in a difference Fourier map, restrained with O—H = 0.89 Å, and refined with *U*_iso_(H)= 1.2 times *U*_eq_(O).

### Crystal data

**Table:**

[Zn_2_(C_10_H_2_O_8_)(C_16_H_20_N_4_)(H_2_O)_2_]	*F*(000) = 700
*M**_r_* = 685.24	*D*_x_ = 1.732 Mg m^−^^3^
Monoclinic, *P*2_1_/*c*	Mo *K*α radiation, λ = 0.71073 Å
*a* = 9.5630 (5) Å	Cell parameters from 2721 reflections
*b* = 9.8747 (5) Å	θ = 3.7–24.2°
*c* = 16.1808 (7) Å	µ = 1.89 mm^−^^1^
β = 120.673 (2)°	*T* = 293 K
*V* = 1314.20 (11) Å^3^	Block, yellow
*Z* = 2	0.22 × 0.16 × 0.15 mm

### Data collection

**Table:**

Bruker SMART APEXII CCD diffractometer	3096 independent reflections
Radiation source: fine-focus sealed tube	2622 reflections with *I* > 2σ(*I*)
graphite	*R*_int_ = 0.027
φ and ω scans	θ_max_ = 28.7°, θ_min_ = 2.5°
Absorption correction: multi-scan (*SADABS*; Bruker, 2004)	*h* = −12→12
*T*_min_ = 0.672, *T*_max_ = 0.767	*k* = −12→6
7908 measured reflections	*l* = −20→20

### Refinement

**Table:**

Refinement on *F*^2^	Primary atom site location: structure-invariant direct methods
Least-squares matrix: full	Secondary atom site location: difference Fourier map
*R*[*F*^2^ > 2σ(*F*^2^)] = 0.028	Hydrogen site location: inferred from neighbouring sites
*wR*(*F*^2^) = 0.071	H-atom parameters constrained
*S* = 1.03	*w* = 1/[σ^2^(*F*_o_^2^) + (0.0309*P*)^2^ + 0.7095*P*] where *P* = (*F*_o_^2^ + 2*F*_c_^2^)/3
3096 reflections	(Δ/σ)_max_ = 0.001
190 parameters	Δρ_max_ = 0.44 e Å^−^^3^
0 restraints	Δρ_min_ = −0.44 e Å^−^^3^

### Special details

**Table:**

Geometry. All e.s.d.'s (except the e.s.d. in the dihedral angle between two l.s. planes) are estimated using the full covariance matrix. The cell e.s.d.'s are taken into account individually in the estimation of e.s.d.'s in distances, angles and torsion angles; correlations between e.s.d.'s in cell parameters are only used when they are defined by crystal symmetry. An approximate (isotropic) treatment of cell e.s.d.'s is used for estimating e.s.d.'s involving l.s. planes.
Refinement. Refinement of *F*^2^ against ALL reflections. The weighted *R*-factor *wR* and goodness of fit *S* are based on *F*^2^, conventional *R*-factors *R* are based on *F*, with *F* set to zero for negative *F*^2^. The threshold expression of *F*^2^ > σ(*F*^2^) is used only for calculating *R*-factors(gt) *etc*. and is not relevant to the choice of reflections for refinement. *R*-factors based on *F*^2^ are statistically about twice as large as those based on *F*, and *R*- factors based on ALL data will be even larger.

### Fractional atomic coordinates and isotropic or equivalent isotropic displacement parameters (Å^2^)

**Table:**

	*x*	*y*	*z*	*U*_iso_*/*U*_eq_	
Zn1	0.86004 (3)	0.65356 (2)	0.795439 (16)	0.02201 (8)	
O1	0.80856 (18)	0.79217 (16)	0.69580 (11)	0.0338 (4)	
O2	0.6272 (2)	0.8500 (2)	0.73459 (13)	0.0557 (6)	
O3	0.91934 (16)	1.09247 (16)	0.66965 (10)	0.0286 (3)	
O4	0.92729 (17)	0.96053 (17)	0.56165 (11)	0.0324 (4)	
O5	0.8690 (2)	0.69929 (17)	0.91493 (11)	0.0420 (4)	
H5A	0.8933	0.6451	0.9611	0.063*	
H5B	0.8548	0.7783	0.9303	0.063*	
N1	0.6991 (2)	0.50031 (18)	0.72410 (12)	0.0259 (4)	
N2	0.14900 (18)	0.44565 (17)	0.51164 (11)	0.0216 (3)	
C1	0.6825 (2)	0.8567 (2)	0.68146 (14)	0.0258 (4)	
C2	0.5924 (2)	0.93592 (19)	0.58884 (13)	0.0188 (4)	
C3	0.6703 (2)	1.00226 (19)	0.54740 (13)	0.0185 (4)	
C4	0.4233 (2)	0.9353 (2)	0.54138 (13)	0.0209 (4)	
H4	0.3718	0.8921	0.5697	0.025*	
C5	0.8530 (2)	1.0158 (2)	0.59632 (14)	0.0213 (4)	
C6	0.7442 (3)	0.3710 (2)	0.74772 (17)	0.0337 (5)	
H6	0.8454	0.3527	0.8014	0.040*	
C7	0.6469 (3)	0.2646 (2)	0.69576 (17)	0.0372 (5)	
H7	0.6810	0.1759	0.7146	0.045*	
C8	0.4979 (3)	0.2906 (2)	0.61535 (16)	0.0320 (5)	
H8	0.4308	0.2194	0.5792	0.038*	
C9	0.4484 (2)	0.4234 (2)	0.58848 (14)	0.0240 (4)	
C10	0.5518 (2)	0.5241 (2)	0.64550 (15)	0.0255 (4)	
H10	0.5188	0.6136	0.6292	0.031*	
C11	0.2899 (2)	0.4555 (2)	0.49833 (14)	0.0277 (5)	
H11A	0.2952	0.5465	0.4775	0.033*	
H11B	0.2747	0.3934	0.4479	0.033*	
C12	0.1496 (2)	0.5559 (2)	0.57275 (14)	0.0259 (4)	
H12A	0.1503	0.6424	0.5445	0.031*	
H12B	0.2473	0.5503	0.6356	0.031*	
C13	−0.0016 (2)	0.4524 (2)	0.41650 (14)	0.0268 (4)	
H13A	−0.0033	0.3782	0.3767	0.032*	
H13B	−0.0039	0.5366	0.3850	0.032*	

### Atomic displacement parameters (Å^2^)

**Table:**

	*U*^11^	*U*^22^	*U*^33^	*U*^12^	*U*^13^	*U*^23^
Zn1	0.01665 (12)	0.02323 (14)	0.01964 (12)	0.00100 (9)	0.00455 (9)	0.00333 (9)
O1	0.0293 (8)	0.0325 (9)	0.0299 (8)	0.0108 (7)	0.0080 (7)	0.0142 (7)
O2	0.0492 (11)	0.0897 (17)	0.0311 (9)	0.0197 (10)	0.0226 (9)	0.0280 (10)
O3	0.0168 (7)	0.0368 (9)	0.0263 (7)	−0.0055 (6)	0.0067 (6)	−0.0122 (6)
O4	0.0217 (7)	0.0422 (10)	0.0313 (8)	−0.0014 (7)	0.0122 (6)	−0.0115 (7)
O5	0.0718 (13)	0.0275 (9)	0.0280 (8)	0.0159 (8)	0.0264 (9)	0.0076 (7)
N1	0.0180 (8)	0.0266 (9)	0.0271 (9)	−0.0008 (7)	0.0072 (7)	0.0006 (7)
N2	0.0143 (7)	0.0277 (9)	0.0188 (8)	−0.0016 (7)	0.0056 (6)	−0.0025 (7)
C1	0.0219 (10)	0.0233 (11)	0.0195 (9)	−0.0036 (8)	0.0013 (8)	0.0034 (8)
C2	0.0170 (9)	0.0163 (9)	0.0162 (8)	0.0006 (7)	0.0034 (7)	0.0009 (7)
C3	0.0137 (8)	0.0167 (9)	0.0184 (9)	−0.0015 (7)	0.0033 (7)	−0.0032 (7)
C4	0.0186 (9)	0.0203 (10)	0.0205 (9)	−0.0038 (7)	0.0075 (8)	0.0028 (7)
C5	0.0156 (9)	0.0222 (10)	0.0205 (9)	−0.0010 (7)	0.0052 (7)	0.0013 (8)
C6	0.0241 (11)	0.0308 (12)	0.0320 (12)	0.0043 (9)	0.0040 (9)	0.0010 (9)
C7	0.0348 (13)	0.0230 (11)	0.0425 (13)	0.0040 (9)	0.0116 (11)	−0.0022 (10)
C8	0.0274 (11)	0.0287 (12)	0.0352 (12)	−0.0047 (9)	0.0125 (10)	−0.0094 (9)
C9	0.0167 (9)	0.0315 (11)	0.0242 (10)	−0.0006 (8)	0.0105 (8)	−0.0016 (8)
C10	0.0198 (10)	0.0245 (11)	0.0288 (10)	0.0000 (8)	0.0100 (8)	0.0020 (8)
C11	0.0176 (9)	0.0394 (13)	0.0238 (10)	−0.0038 (9)	0.0089 (8)	−0.0019 (9)
C12	0.0160 (9)	0.0323 (12)	0.0229 (10)	−0.0044 (8)	0.0052 (8)	−0.0075 (8)
C13	0.0189 (9)	0.0361 (12)	0.0189 (9)	−0.0002 (9)	0.0049 (8)	−0.0057 (8)

### Geometric parameters (Å, °)

**Table:**

Zn1—O5	1.9442 (16)	C3—C5	1.512 (2)
Zn1—O1	1.9762 (15)	C4—C3^iii^	1.390 (3)
Zn1—O3^i^	1.9795 (14)	C4—H4	0.9300
Zn1—N1	2.0443 (18)	C6—C7	1.370 (3)
O1—C1	1.276 (3)	C6—H6	0.9300
O2—C1	1.220 (3)	C7—C8	1.377 (3)
O3—C5	1.271 (2)	C7—H7	0.9300
O3—Zn1^ii^	1.9795 (14)	C8—C9	1.387 (3)
O4—C5	1.235 (2)	C8—H8	0.9300
O5—H5A	0.8501	C9—C10	1.374 (3)
O5—H5B	0.8501	C9—C11	1.507 (3)
N1—C6	1.340 (3)	C10—H10	0.9300
N1—C10	1.352 (3)	C11—H11A	0.9700
N2—C12	1.469 (3)	C11—H11B	0.9700
N2—C11	1.472 (2)	C12—C13^iv^	1.514 (3)
N2—C13	1.479 (2)	C12—H12A	0.9700
C1—C2	1.511 (3)	C12—H12B	0.9700
C2—C4	1.392 (3)	C13—C12^iv^	1.514 (3)
C2—C3	1.394 (3)	C13—H13A	0.9700
C3—C4^iii^	1.390 (3)	C13—H13B	0.9700
			
O5—Zn1—O1	121.11 (7)	N1—C6—H6	118.8
O5—Zn1—O3^i^	106.68 (7)	C7—C6—H6	118.8
O1—Zn1—O3^i^	103.27 (6)	C6—C7—C8	119.3 (2)
O5—Zn1—N1	111.84 (8)	C6—C7—H7	120.4
O1—Zn1—N1	104.45 (7)	C8—C7—H7	120.4
O3^i^—Zn1—N1	108.74 (7)	C7—C8—C9	119.7 (2)
C1—O1—Zn1	108.28 (14)	C7—C8—H8	120.2
C5—O3—Zn1^ii^	113.58 (13)	C9—C8—H8	120.2
Zn1—O5—H5A	125.6	C10—C9—C8	117.44 (19)
Zn1—O5—H5B	124.8	C10—C9—C11	121.47 (19)
H5A—O5—H5B	109.5	C8—C9—C11	121.07 (19)
C6—N1—C10	117.61 (18)	N1—C10—C9	123.6 (2)
C6—N1—Zn1	120.29 (14)	N1—C10—H10	118.2
C10—N1—Zn1	121.67 (14)	C9—C10—H10	118.2
C12—N2—C11	111.10 (16)	N2—C11—C9	112.87 (16)
C12—N2—C13	109.56 (15)	N2—C11—H11A	109.0
C11—N2—C13	108.82 (15)	C9—C11—H11A	109.0
O2—C1—O1	123.6 (2)	N2—C11—H11B	109.0
O2—C1—C2	119.6 (2)	C9—C11—H11B	109.0
O1—C1—C2	116.57 (19)	H11A—C11—H11B	107.8
C4—C2—C3	119.48 (17)	N2—C12—C13^iv^	110.75 (16)
C4—C2—C1	117.36 (17)	N2—C12—H12A	109.5
C3—C2—C1	123.07 (17)	C13^iv^—C12—H12A	109.5
C4^iii^—C3—C2	119.02 (17)	N2—C12—H12B	109.5
C4^iii^—C3—C5	117.67 (17)	C13^iv^—C12—H12B	109.5
C2—C3—C5	123.24 (17)	H12A—C12—H12B	108.1
C3^iii^—C4—C2	121.50 (18)	N2—C13—C12^iv^	110.43 (16)
C3^iii^—C4—H4	119.3	N2—C13—H13A	109.6
C2—C4—H4	119.3	C12^iv^—C13—H13A	109.6
O4—C5—O3	123.94 (18)	N2—C13—H13B	109.6
O4—C5—C3	120.13 (17)	C12^iv^—C13—H13B	109.6
O3—C5—C3	115.80 (17)	H13A—C13—H13B	108.1
N1—C6—C7	122.4 (2)		
			
O5—Zn1—O1—C1	−48.49 (17)	C4^iii^—C3—C5—O4	−67.8 (3)
O3^i^—Zn1—O1—C1	−167.62 (14)	C2—C3—C5—O4	115.2 (2)
N1—Zn1—O1—C1	78.70 (15)	C4^iii^—C3—C5—O3	108.3 (2)
O5—Zn1—N1—C6	−83.24 (18)	C2—C3—C5—O3	−68.7 (3)
O1—Zn1—N1—C6	144.06 (17)	C10—N1—C6—C7	−0.3 (4)
O3^i^—Zn1—N1—C6	34.32 (19)	Zn1—N1—C6—C7	−173.04 (19)
O5—Zn1—N1—C10	104.37 (17)	N1—C6—C7—C8	1.0 (4)
O1—Zn1—N1—C10	−28.33 (18)	C6—C7—C8—C9	−0.3 (4)
O3^i^—Zn1—N1—C10	−138.07 (16)	C7—C8—C9—C10	−1.0 (3)
Zn1—O1—C1—O2	13.3 (3)	C7—C8—C9—C11	177.0 (2)
Zn1—O1—C1—C2	−161.64 (13)	C6—N1—C10—C9	−1.0 (3)
O2—C1—C2—C4	−34.3 (3)	Zn1—N1—C10—C9	171.57 (16)
O1—C1—C2—C4	140.9 (2)	C8—C9—C10—N1	1.7 (3)
O2—C1—C2—C3	149.1 (2)	C11—C9—C10—N1	−176.32 (19)
O1—C1—C2—C3	−35.7 (3)	C12—N2—C11—C9	70.1 (2)
C4—C2—C3—C4^iii^	−0.9 (3)	C13—N2—C11—C9	−169.16 (18)
C1—C2—C3—C4^iii^	175.55 (18)	C10—C9—C11—N2	−100.7 (2)
C4—C2—C3—C5	176.05 (18)	C8—C9—C11—N2	81.4 (2)
C1—C2—C3—C5	−7.5 (3)	C11—N2—C12—C13^iv^	178.22 (17)
C3—C2—C4—C3^iii^	1.0 (3)	C13—N2—C12—C13^iv^	57.9 (2)
C1—C2—C4—C3^iii^	−175.72 (18)	C12—N2—C13—C12^iv^	−57.8 (2)
Zn1^ii^—O3—C5—O4	14.7 (3)	C11—N2—C13—C12^iv^	−179.41 (18)
Zn1^ii^—O3—C5—C3	−161.19 (13)		

Symmetry codes: (i) −*x*+2, *y*−1/2, −*z*+3/2; (ii) −*x*+2, *y*+1/2, −*z*+3/2; (iii) −*x*+1, −*y*+2, −*z*+1; (iv) −*x*, −*y*+1, −*z*+1.

### Hydrogen-bond geometry (Å, °)

**Table:**

*D*—H···*A*	*D*—H	H···*A*	*D*···*A*	*D*—H···*A*
O5—H5A···O4^v^	0.85	1.82	2.662 (2)	173
O5—H5B···N2^vi^	0.85	1.91	2.751 (2)	169

Symmetry codes: (v) *x*, −*y*+3/2, *z*+1/2; (vi) −*x*+1, *y*+1/2, −*z*+3/2.
